# Obesity's Unexpected Influence: Reduced Alphavirus Transmission and Altered Immune Activation in the Vector

**DOI:** 10.1002/jmv.70032

**Published:** 2024-10-28

**Authors:** Pallavi Rai, Emily M. Webb, Sally L. Paulson, Lin Kang, James Weger‐Lucarelli

**Affiliations:** ^1^ Department of Biomedical Sciences and Pathobiology Virginia Tech Blacksburg Virginia USA; ^2^ Center for Emerging, Zoonotic, and Arthropod‐Borne Pathogens Virginia Tech Blacksburg Virginia USA; ^3^ Department of Entomology Fralin Life Sciences Institute, Virginia Tech Blacksburg Virginia USA; ^4^ Biomedical Research Edward Via College of Osteopathic Medicine Monroe Los Angeles USA; ^5^ College of Pharmacy University of Louisiana Monroe Monroe Los Angeles USA

**Keywords:** *Aedes aegypti* mosquitoes, alphavirus, chikungunya virus, gene silencing, Mayaro virus, obesity

## Abstract

Chikungunya virus (CHIKV) and Mayaro virus (MAYV) are emerging/re‐emerging alphaviruses transmitted by *Aedes* spp. mosquitoes and responsible for recent disease outbreaks in the Americas. The capacity of these viruses to cause epidemics is frequently associated with increased mosquito transmission, which in turn is governed by virus−host−vector interactions. Although many studies have explored virus−vector interactions, significant gaps remain in understanding how vertebrate host factors influence alphavirus transmission by mosquitoes. We previously showed that obesity, a ubiquitous vertebrate host biological factor, reduces alphavirus transmission potential in mosquitoes. We hypothesized that alphavirus‐infected obese bloodmeals altered immune genes and/or pathways in mosquitoes, thereby inhibiting virus transmission. To test this, we conducted RNA sequencing (RNA‐seq) and reverse transcription‐quantitative polymerase chain reaction (RT‐qPCR) on midgut RNA from mosquitoes fed on alphavirus‐infected lean and obese mice. This approach aimed to identify potential antiviral or proviral genes and pathways altered in mosquitoes after consuming infected obese bloodmeals. We found upregulation of the Toll pathway and downregulation of several metabolic and other genes in mosquitoes fed on alphavirus‐infected obese bloodmeals. Through gene knockdown studies, we demonstrated the antiviral role of Toll pathway and proviral roles of AAEL009965 and fatty acid synthase (FASN) in the transmission of alphaviruses by mosquitoes. Therefore, this study utilized obesity to identify factors influencing alphavirus transmission by mosquitoes and this research approach may pave the way for designing broadly effective antiviral measures to combat mosquito‐borne viruses, such as releasing transgenic mosquitoes deficient in the identified genes.

## 1 Introduction

Chikungunya virus (CHIKV) and Mayaro virus (MAYV) are emerging/re‐emerging mosquito‐borne alphaviruses that have caused recent outbreaks of acute febrile illness and chronic debilitating polyarthritis [1, 2, 3] in the Americas. According to the Pan American Health Organization (PAHO), during the first quarter of 2024, approximately 186 000 cases of CHIKV were reported in the Americas [4], with the first reported local transmission recorded in Argentina and Uruguay [5]. First identified in Trinidad and Tobago in 1954 [6], MAYV is now spreading throughout the Americas [7], but its true prevalence may be underreported due to the absence of specific diagnostics and clinical symptoms [8] that overlap with those of other important mosquito‐borne diseases, such as dengue, malaria, and chikungunya. The lack of widely available vaccines and treatments, combined with the widespread distribution of competent vectors, such as *Aedes aegypti* and *Aedes albopictus* [9, 10, 11], underscores the substantial public health risk and epidemic potential posed by CHIKV and MAYV.

The epidemic potential of these viruses is frequently related to their enhanced transmission [12] by the mosquito vector defined by vector competence, which is the ability of a vector to successfully transmit a pathogen [13]. While extensive research has demonstrated the influence of tissue barriers and antiviral responses within mosquitoes on their vector competence for various mosquito‐borne viruses [14, 15, 16, 17, 18, 19, 20, 21, 22], studies to investigate the impact of vertebrate host factors on the vector and their role in influencing the transmission potential of various viruses, remain limited. Obesity now considered a global pandemic [23] affecting 13% of adults worldwide and 42% of adults in the United States [24], has been shown to worsen outcomes for various important human pathogens, including severe acute respiratory syndrome coronavirus 2 (SARS‐CoV‐2) [25], influenza virus [26], and as our research has shown, mosquito‐borne viruses such as CHIKV, MAYV [27], and dengue virus (DENV) [28]. Interestingly, we also found that *Ae. aegypti* mosquitoes fed on diet‐induced obese (hereinafter referred to as obese) mice infected with MAYV exhibited lower infection and transmission rates compared to those fed on healthy weight (hereinafter referred to as lean) mice, despite similar levels of viremia between both the groups [27]. However, the mechanism by which alphavirus‐infected obese bloodmeal impacts their transmission by mosquitoes is still unknown.

In this study, we used a natural transmission cycle using mosquitoes and alphavirus‐infected lean and obese mice and confirmed our previous finding that feeding on alphavirus‐infected obese bloodmeal reduced their transmission by the mosquitoes. To determine the impact of alphavirus‐infected obese bloodmeal on mosquito gene expression, we performed RNA sequencing (RNA‐seq) on midguts collected from mosquitoes fed on CHIKV‐ or MAYV‐infected lean or obese mice at 1‐ and 4‐days‐post‐infectious‐bloodmeal (dpbm). We identified activation of the Toll pathway and downregulation of several metabolic and other genes at 1‐dpbm in mosquitoes fed on CHIKV‐ or MAYV‐infected obese mice compared to those fed on their lean counterparts. Through these studies, we have utilized obesity to identify two genes, AAEL009965 and fatty acid synthase (FASN), and validated their proviral role in CHIKV and MAYV infections through knockdown studies in mosquitoes. We have also shown the importance of the Toll pathway in controlling alphavirus infection in mosquitoes. These findings could lay the groundwork for developing broadly effective antiviral strategies against major mosquito‐borne viruses.

## 2 Methods

### 2.1 Biohazard Considerations

All MAYV experiments were conducted in a BSL‐2 facility, while CHIKV studies were carried out in a BSL‐3 facility in compliance with all CDC and NIH guidelines. These studies were approved by the Institutional Animal Care and Use Committee (IACUC#21‐041) and Institutional Biosafety Committee (IBC#21‐084) at Virginia Tech. All mosquito studies were conducted under ACL‐3 conditions.

### 2.2 Cells and Viruses

Vero cells (CCL‐81) purchased from American Type Culture Collection (ATCC; Manassas, VA), were cultured in Dulbecco's Modified Eagle's Medium (DMEM) supplemented with 5% fetal bovine serum (FBS), 1 mg/mL gentamicin, 1% non‐essential amino acids (NEAAs), and 25 mM HEPES buffer in an incubator at 37°C with 5% CO_2_. CHIKV SL‐15649 (ECSA lineage), derived from an infectious clone, was kindly provided by Dr. Mark Heise [29] (the University of North Carolina at Chapel Hill). MAYV strain TRVL4675 used for this study was derived from an infectious clone and has been described previously [30]. Infectious viral titers were determined by plaque assay as previously described [31].

### 2.3 Mice and Diets

C57BL/6N mice of both sexes, obtained from Charles River Laboratories at 3−4 weeks of age, were allowed a 1‐week acclimation period before initiating diets. Mice were housed in groups of 3−5 per cage and maintained at ambient temperature with access to food and water ad libitum. All diets used for the study were obtained from Research Diets (New Brunswick, NJ, USA). Twenty‐eight mice were placed on a low‐fat diet with 10% kcal fat (LFD; D12450K) and 32 mice on a high‐fat diet with 60% kcal fat (HFD; D12492). Throughout the manuscript, we refer to the groups as lean (LFD) or obese (HFD). The mice were placed on their respective diets 16−18 weeks before infections and remained on the diets until the end of the experiments.

### 2.4 Glucose and Insulin Measurements

Two weeks before infections (16 weeks after diet initiation), mice were bled via the submandibular vein, and non‐fasting glucose levels were measured at 9−10 a.m. using the Abox glucose monitoring kit. Serum insulin levels were quantified using the Mouse Insulin ELISA kit (Mercodia, NC, USA) according to the manufacturer's instructions. A standard curve was generated and used to calculate the insulin levels of each sample, expressed in µg/L.

### 2.5 Mouse Infections

Mice were inoculated subcutaneously in the left hind footpads, as described previously [32]. For CHIKV and MAYV infections, 10^2^ and 10^4^ plaque‐forming units (PFUs) of the virus in 50 µL of Roswell Park Memorial Institute (RPMI)‐1640 media with no additives were used, respectively. Mice were weighed daily and monitored for weight loss. Blood was collected via the submandibular vein, and serum was separated by centrifugation at 5000×*g* for 10 min, transferred to fresh tubes, and stored at ‐80°C. For infections and blood collection, mice were anesthetized using isoflurane inhalation. Mice were treated with 0.1 mg in 100 μL of IFNAR1 blocking antibody (MAR1‐5A3, Leinco Technologies Inc., Missouri, USA) via the intraperitoneal route 1 day before CHIKV infection to increase their susceptibility to infection [33]. Viremia was measured in all studies by plaque assay as previously described.

### 2.6 Mosquito Transmission Studies

*Ae. aegypti* females from the 20th to 24th generation of a colony derived from wild mosquitoes collected from Guerrero (Mexico) [34] were used for these studies. The mosquitoes were maintained on a 12 h light:12 h dark cycle, at 28°C with 70% relative humidity (RH) and fed with 10% sucrose solution ad libitum. When the mice had high viremia—2 days for MAYV and 2 or 3 days for CHIKV—the mice were anesthetized with ketamine and xylazine (90 and 5 mg/kg, respectively) intraperitoneally, and the mosquitoes were allowed to feed for 45 min. Fully engorged mosquitoes were sorted into new cages and maintained in an environmental chamber under the conditions mentioned above. Immediately after the bloodmeal, 10−15 fully engorged mosquitoes were placed individually in 2 mL tubes containing a metal bead and mosquito diluent (RPMI media with 2% FBS, 10 mM HEPES, 50 μg/mL of gentamicin sulfate, and 2.5 μg/mL of Amphotericin B) for comparing viral titers in mosquitoes fed on infected lean and obese mice as described below. A subset of 10−20 mosquitoes was kept in separate cartons for both CHIKV & MAYV, to perform midgut dissections at 1‐ and 4‐dpbm to determine immune pathway activation in the vector in response to alphavirus‐infected obese bloodmeal.

After 9 days, the remaining mosquitoes were coldly anesthetized and bodies, legs/wings, and saliva were collected to assess infection, dissemination, and transmission, respectively. As previously described, bodies and legs/wings were homogenized using Qiagen's TissueLyzer II [35]. Saliva was collected by placing the proboscis in a 20 μL barrier tip containing 10 μL immersion oil for 45 min, after which the contents were pipetted into a tube containing 50 μL mosquito diluent. Saliva samples and the supernatant from bodies and legs/wings were tested for infectious virus by plaque assays [31].

### 2.7 RNA Extraction From Mosquito Midguts

Mosquito midguts were dissected 1‐ and 4‐dpbm and stored in 1.7 mL tubes containing 200 µL TRIzol reagent (ThermoFisher). RNA was extracted per the manufacturer's protocol and stored at −80°C until use.

### 2.8 Reverse Transcription‐Quantitative Polymerase Chain Reaction (RT‐qPCR)

All primers were ordered from Integrated DNA Technologies (IDT, Iowa, USA) and are listed in Supporting Information S1: Table S1. RT‐qPCR was performed on midgut RNA isolated from mosquitoes fed on CHIKV‐ or MAYV‐infected lean and obese mice using the NEB Luna Universal One‐Step‐RT‐qPCR kit with SYBR Green (NEB, MA, USA). The reaction conditions in a Biorad CFX‐96 were reverse transcription at 55°C for 10 min and initial denaturation and reverse transcriptase deactivation at 95°C for 1 min, followed by 44 cycles of denaturation at 95°C for 10 s and 60°C for 30 s for annealing/extension. A melt curve was generated from 60°C to 95°C with 0.5°C increments at an interval of 0.05 s. For comparing relative gene expression, the expression of the genes of interest (GOI) were normalized to *Rp49*, a housekeeping gene that has been used for *Drosophila* [36], live mosquitoes [37], and other insects [38].

### 2.9 Analysis of RNA‐Seq Data and Pathway Enrichment Analysis

RNA extracted from the midguts of mosquitoes fed on CHIKV‐ or MAYV‐infected lean and obese mice were submitted to Novogene Corporation Inc. (Sacramento, CA, USA) for library preparation and RNA seq using NovaSeq PE150 sequencing platform. The average sequencing bases per sample was 9667.37 Mbp with Q30 of 96.38%. Raw reads were quality‐controlled using FastQC [39] and trimmed with BBDuk [40]. The reference genome (AaegL5) and corresponding annotations were downloaded from Vectorbase [41]. The clean reads were mapped to the reference genome using STAR [42] with default parameters. Gene counts were estimated using HT‐Seq (htseq‐count) [43]. Differentially expressed genes (DEGs) with a fold change threshold of > 3 were identified using DESeq. 2 [44]. Pathway analysis on these DEGs was conducted using the integrated differential expression and pathway analysis tool (iDEP.96, South Dakota State University, SD) [45].

### 2.10 Double‐Stranded RNA (dsRNA) Synthesis

dsRNAs for performing gene knockdown studies were designed and synthesized as described previously [46]. The GOI were PCR amplified using gene‐specific primers with the T7 promoter sequence at the 5’ end of both primers. dsRNA targeting the green fluorescent protein (GFP) was used as a control. All primers were ordered from Integrated DNA Technologies (IDT, Iowa, USA) and are listed in Supporting Information S1: Table S2. The template for GFP was a GFP‐containing plasmid, while RNA isolated from a pool of *Ae. aegypti* mosquitoes was used as a template for GOI. Purified PCR amplicons were used as a template for dsRNA synthesis using the MEGAscript T7 transcription kit (ThermoFisher) according to manufacturer guidelines.

### 2.11 Gene Silencing and Infections Post‐Silencing in *Ae. aegypti* Mosquitoes

RNAi‐mediated gene silencing was performed as previously described [46]. Briefly, 1−2‐day‐old *Ae. aegypti* mosquitoes were coldly anesthetized and then intrathoracically (IT) injected with 500 ng of dsRNA targeting either the GOI or GFP using the Nanoject II system (Drummond). For Myd88 knockdowns, 200 ng of dsRNA was used [47]. After injection, mosquitoes were kept in an environmental chamber for 3 days at 28°C and 70%−80% RH and fed with 10% sucrose solution ad libitum. Knockdown efficiency was tested 3‐days‐post‐IT (dpIT) injections using the delta−delta *C*_t_ (ΔΔ*C*_t_) relative quantification method [48], with average GFP values used as control and *Rp49* as the housekeeping gene.

Mosquitoes were sucrose and water‐starved on Day 2‐dpIT and exposed to an infectious blood meal containing CHIKV or MAYV at 3‐dpIT. For infections, mosquitoes were exposed to an artificial blood meal containing defibrinated sheep's blood and 7 × 10^7^ PFUs/mL of CHIKV [49] or MAYV using a Hemotek membrane feeder. For artificial infections, the infectious blood meal comprised 30% CHIKV virus mixed with 70% defibrinated sheep blood, while for MAYV, equal volumes of the virus and sheep blood were used. Natural transmission studies were performed with mosquitoes knocked down for Myd88 or GFP and exposed to MAYV‐infected obese mice at peak viremia (2 dpi). Fully engorged mosquitoes were sorted and maintained in an environmental chamber under the conditions mentioned above. After 9 days, the mosquitoes were coldly anesthetized and bodies, legs/wings, and saliva were collected and tested for infection, dissemination, and transmission, respectively, as described above.

### 2.12 Statistical Analysis

Statistical and regression analyses were conducted using GraphPad Prism 9 (GraphPad Software, San Diego, CA, USA). Details of the statistical technique used for the analyses are provided in the figure legends. The level of significance has been determined by the following *p*‐values: *p* ≥ 0.05 (ns); *p = 0.05 to 0.01; **p = 0.01 to 0.001; ***p = 0.001 to 0.0001; ****p < 0.0001. Error bars represent the standard deviation from the mean, and the dotted lines denote the limit of detection (LOD). All statistical analyses were conducted on data that were first tested for normality using the Shapiro−Wilk test and for outliers using the ROUT method.

## 3 Results

### 3.1 C57BL/6N Mice Fed a HFD Developed Hyperglycemia and Hyperinsulinemia, Resembling Obesity in Humans

**Figure 1:**
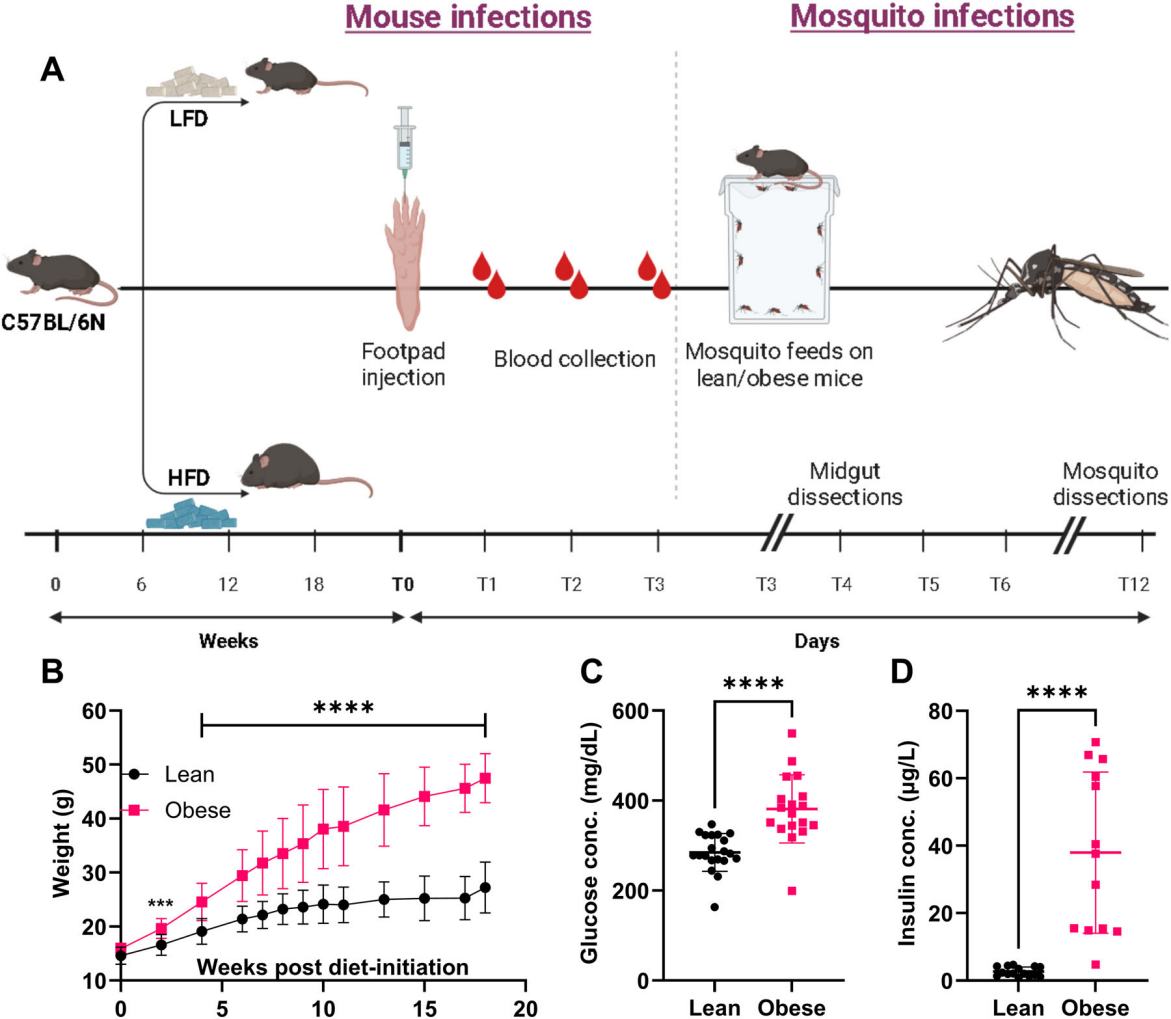
C57BL/6N mice fed a high‐fat diet develop diet‐induced obesity (DIO) with hyperglycemia and hyperinsulinemia resembling humans with obesity. (A) Experimental design. Four‐week‐old C57BL/6N were fed a high (60% fat) or a low (10% fat) diet for 18 weeks. Lean and obese C57BL/6N mice (*n* = 8−10/group) were inoculated with either 10^2^ PFUs of CHIKV or 10^4^ PFUs of MAYV via footpad inoculation. For CHIKV infections, the mice were intraperitoneally injected with 0.1 mg of MAR‐1, a type I interferon (IFN) receptor‐blocking antibody, 12 h before infections to make them susceptible to CHIKV. During high viremia (2 dpi for MAYV and 2 or 3 dpi for CHIKV), 3−4 days old allowed to feed on these mice. Midgut dissections were done on 1‐ and 4‐days‐post‐infectious bloodmeal (dpbm), and dissections for vector competence were done at 9‐dpbm. (B) Weights were measured throughout the feeding period at 1−2‐week intervals, and statistical analysis was conducted using mixed‐effects analysis with Sidak's multiple comparisons test. (C) Glucose and (D) insulin levels were measured 2 weeks before infection (16 weeks post‐diet‐initiation). Statistical analysis was done using an unpaired *t*‐test or Mann−Whitney test; ****p < 0.0001. The error bars indicate the standard deviation (SD) of the mean.

We have previously established that obesity worsens the disease outcome following infection with various alphaviruses; however, it also reduces alphavirus infection in mosquitoes [27]. To explore the mechanism by which obesity was driving reduced alphavirus transmission by the vector, we used previously established mouse models of diet‐induced obesity (DIO) [50]. Lean and obese C57BL/6N mice were inoculated subcutaneously with CHIKV or MAYV via footpad injections, and mosquitoes were allowed to feed on these mice at peak viremia. Midgut dissections were performed at 1‐ and 4‐dpbm, and vector competence dissections were conducted at 9‐dpbm (Figure 1A). As expected, obese mice weighed more than the lean mice starting from 2 weeks post‐diet‐initiation and for the remainder of the study (Figure 1B, ****p < 0.0001). Obesity is associated with increased glucose levels (hyperglycemia) [51] and elevated fasting and non‐fasting insulin levels (hyperinsulinemia) [52]. Accordingly, we measured glucose and insulin levels in our lean and obese mice at 16 weeks post‐diet‐initiation, which were significantly higher in obese mice compared to lean mice (Figure 1C,D; ****p < 0.0001). Thus, our mouse model of DIO mimics human obesity in terms of high weight gain, hyperglycemia, and hyperinsulinemia.

### 3.2 *Ae. aegypti* mosquitoes Fed on Alphavirus‐Infected Obese Mice Have Reduced Transmission Potential Despite Ingesting Similar Amounts of Virus

**Figure 2:**
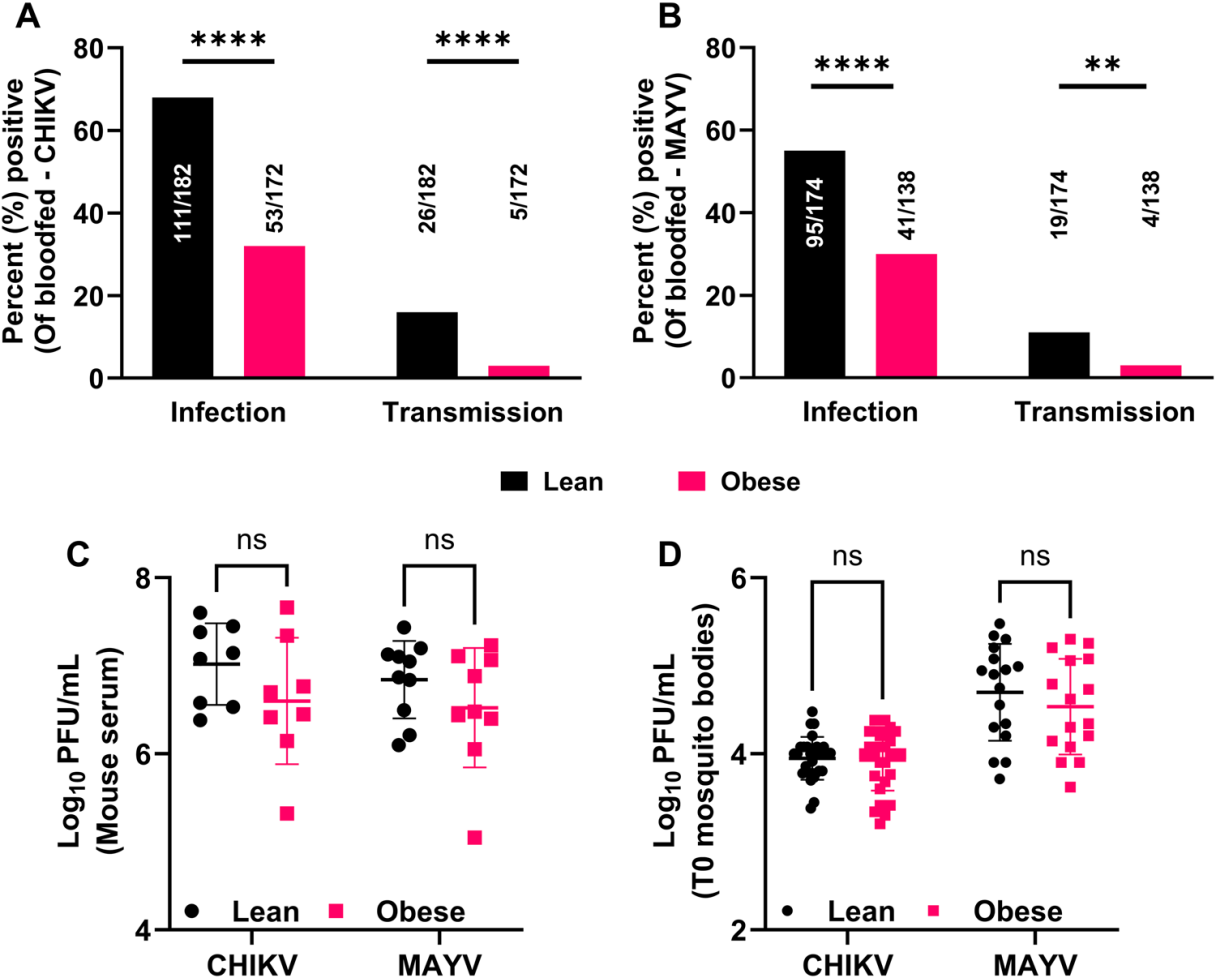
*Aedes aegypti* mosquitoes fed on alphavirus‐infected obese mice have reduced transmission potential. obesity‐associated factor(s). *Ae. aegypti* mosquitoes (*n* = 50−60/mouse) were allowed to feed on CHIKV‐ or MAYV‐infected lean and obese mice at peak viremia. (A, B) Infection and transmission potential of *Ae. aegypti* mosquitoes fed on alphavirus‐infected lean and obese mice for CHIKV (A) and MAYV (B). Infection and transmission rates were calculated as a percentage of samples positive for infectious virus in the bodies and saliva respectively, compared to the total number of bloodfed mosquitoes. Statistical analysis was conducted using a two‐sided Fisher's exact test. (C) Viremia in lean and obese mice before mosquito feeds. On the day of peak viremia, mice were anesthetized with ketamine and xylazine (90 and 5 mg/kg, respectively) intraperitoneally, blood was collected immediately before the mosquito feeds, and infectious virus was quantified by plaque assay. Statistical analysis was performed on log‐transformed values using an unpaired *t*‐test or Mann−Whitney test. (D) Viral titers in mosquito bodies immediately after the blood meal. Mosquitoes were allowed to feed on infected and anesthetized mice for 45 min, after which fully engorged mosquitoes were sorted. Two to three fully engorged mosquitoes were collected from each mouse immediately after the bloodmeals in 2 mL tubes with metal beads and viral diluent. The bodies were homogenized, and infectious viral titers were determined in the supernatant of 20−30 mosquitoes using plaque assays. Statistical analysis was performed on log‐transformed values using unpaired *t*‐test or Mann−Whitney test; **p = 0.0080; ****p < 0.0001; ns, nonsignificant. The error bars indicate the standard deviation (SD) of the mean.

We previously showed that mosquitoes fed on MAYV‐infected obese mice exhibited reduced infection and transmission rates compared to those fed on lean mice [27]. In this study, using a natural transmission cycle between mice and mosquitoes (Figure 1A), we similarly found that mosquitoes fed on infected obese mice had significantly lower infection and transmission rates compared to those fed on lean mice for CHIKV (Figure 2A, ****p < 0.0001 and **p = 0.0011, respectively) and MAYV (Figure 2B, ****p < 0.0001 and **p = 0.0080, respectively). To confirm that the mosquitoes were exposed to similar amounts of virus, we measured viral titers in the serum of alphavirus‐infected lean and obese mice immediately before the mosquito feeding. Our findings indicated no significant differences between the groups for either CHIKV or MAYV (Figure 2C). Additionally, we collected mosquito bodies immediately after they fed on CHIKV‐ and MAYV‐infected mice and quantified infectious virus levels. The results showed similar viral titers (Figure 2D), suggesting that the reduced transmission potential observed in mosquitoes for CHIKV and MAYV was not from the differences in viremia but due to some obesity‐associated factor(s).

### 3.3 The Toll Pathway Is Activated in Mosquito Midguts in Response to Alphavirus‐Infected Obese Bloodmeal

**Figure 3:**
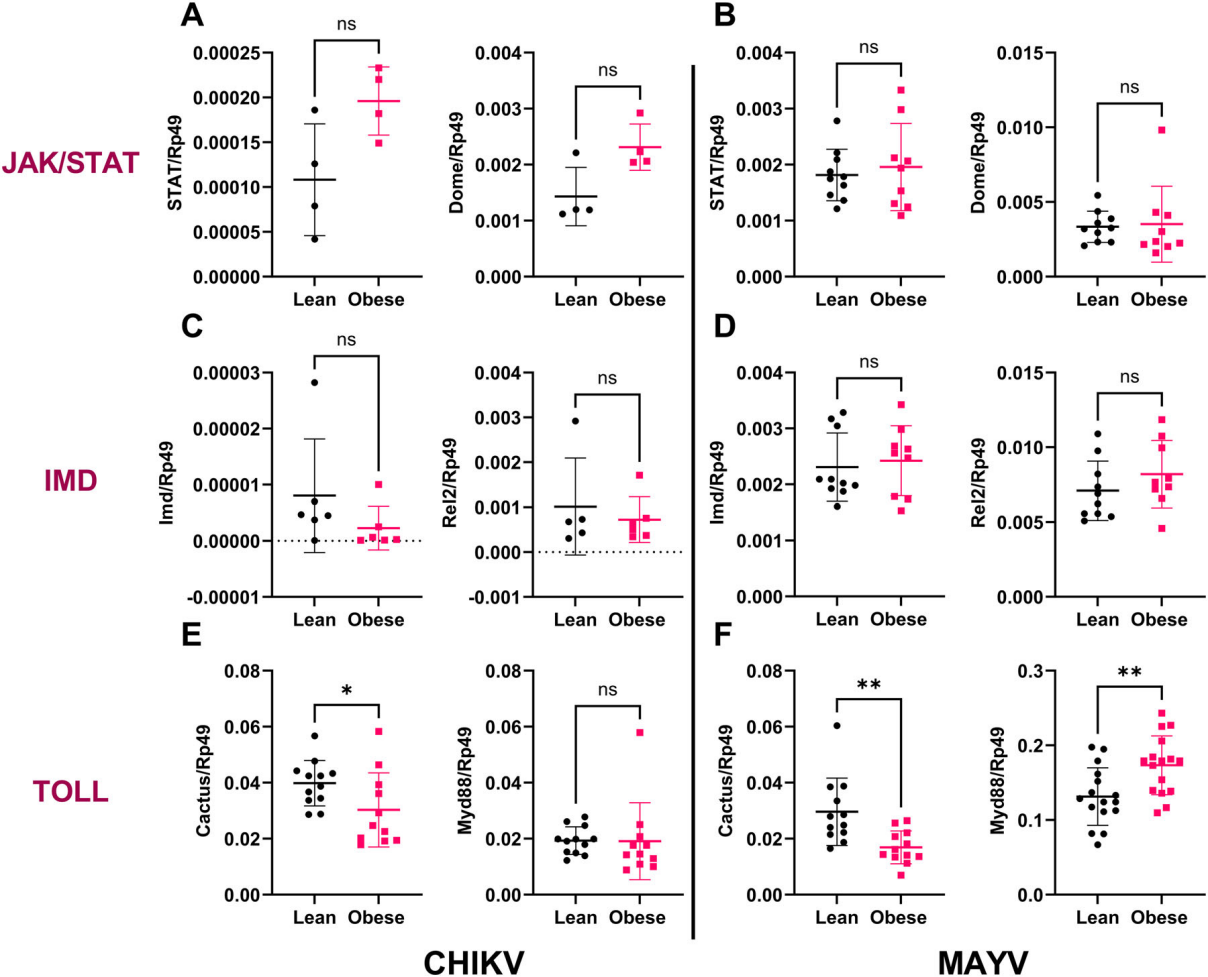
Toll pathway is activated in the midguts of *Aedes aegypti* mosquitoes in response to alphavirus‐infected obese bloodmeal. *Ae. aegypti* mosquitoes (*n* = 50−60/mouse) were allowed to feed on CHIKV‐ or MAYV‐infected lean and obese mice, at peak viremia. Fully engorged mosquitoes were sorted and maintained in an environmental chamber at 28°C with 70%−80% relative humidity, for 9 days. A subset of 10−20 mosquitoes was kept in separate cartons for both CHIKV & MAYV, to perform midgut dissections at 1‐ and 4‐dpbm. Midguts were dissected, RNA was extracted, and gene expression was assessed by reverse‐transcription quantitative PCR (RT‐qPCR). Expression of genes of interest was normalized to the housekeeping gene, Rp49, and then compared between mosquitoes fed on CHIKV‐ or MAYV‐infected lean or obese mice. (A, B) JAK/STAT pathway. The transcription factor, STAT, and the pathway receptor, Domeless (Dome) were tested for mosquitoes (5−10 mosquitoes/group) fed on CHIKV (A) or MAYV (B) infected lean or obese mice. (C, D) IMD pathway. The receptor protein, Imd, and the transcription factor, Rel2 were tested for mosquitoes (5−10 mosquitoes/group) fed on CHIKV (C) or MAYV (D) infected lean or obese mice. (E, F) Expression of the adapter protein, myeloid differentiation primary response (Myd88), and the negative regulator of Toll pathway, cactus were tested for mosquitoes (11−12 mosquitoes/group) fed on CHIKV (E) or MAYV (F) infected lean or obese mice. Statistical analysis was done by unpaired *t*‐test or Mann−Whitney test; *p < 0.01; **p = 0.01 to 0.001; ns, nonsignificant. The error bars indicate the standard deviation (SD) of the mean.

Based on the result that mosquitoes fed on alphavirus‐infected lean or obese mice were exposed to similar amounts of virus, we hypothesized that an alphavirus‐infected obese bloodmeal might modulate the expression of immune‐related genes and pathways in the vector, contributing to reduced transmission. Midgut samples were tested for the major antiviral immune pathways in *Ae. aegypti*, including RNA interference (RNAi), Janus‐kinase signal transducer and activator of transcription (JAK/STAT), Toll, and immunodeficiency (IMD) pathways (Figure 3). For the RNAi pathway, we tested for the expression of argonaute‐2 (Ago‐2), an RNA nuclease critical for RNAi‐mediated antiviral responses, and found no difference between the groups (Supporting Information S1: Figure S1). For the JAK/STAT pathway, we tested the expression of the transcription factor, STAT, and the receptor, Domeless (Dome), and found no difference between mosquitoes fed on CHIKV (Figure 3A) or MAYV (Figure 3B) infected lean or obese mice. Similarly, for the IMD pathway, no difference in the expression of the receptor protein, Imd, or the transcription factor, Rel2, was observed between mosquitoes fed on CHIKV (Figure 3C) or MAYV (Figure 3D) infected lean or obese mice. For the Toll pathway, we evaluated the expression of the adapter protein, Myeloid differentiation primary response 88 (Myd88), and the negative regulator of the Toll pathway, Cactus. We found significant downregulation of Cactus (Figure 3E; *p = 0.0478) while no significant difference in Myd88 expression (Figure 3E) in mosquitoes fed on CHIKV‐infected obese versus lean mice. Similarly, mosquitoes fed on MAYV‐infected obese mice showed a significant downregulation of Cactus (Figure 3F; **p = 0.0011) and an upregulation of Myd88 (Figure 3F; **p = 0.0048) compared to mosquitoes fed on MAYV‐infected lean mice.

**Figure 4:**
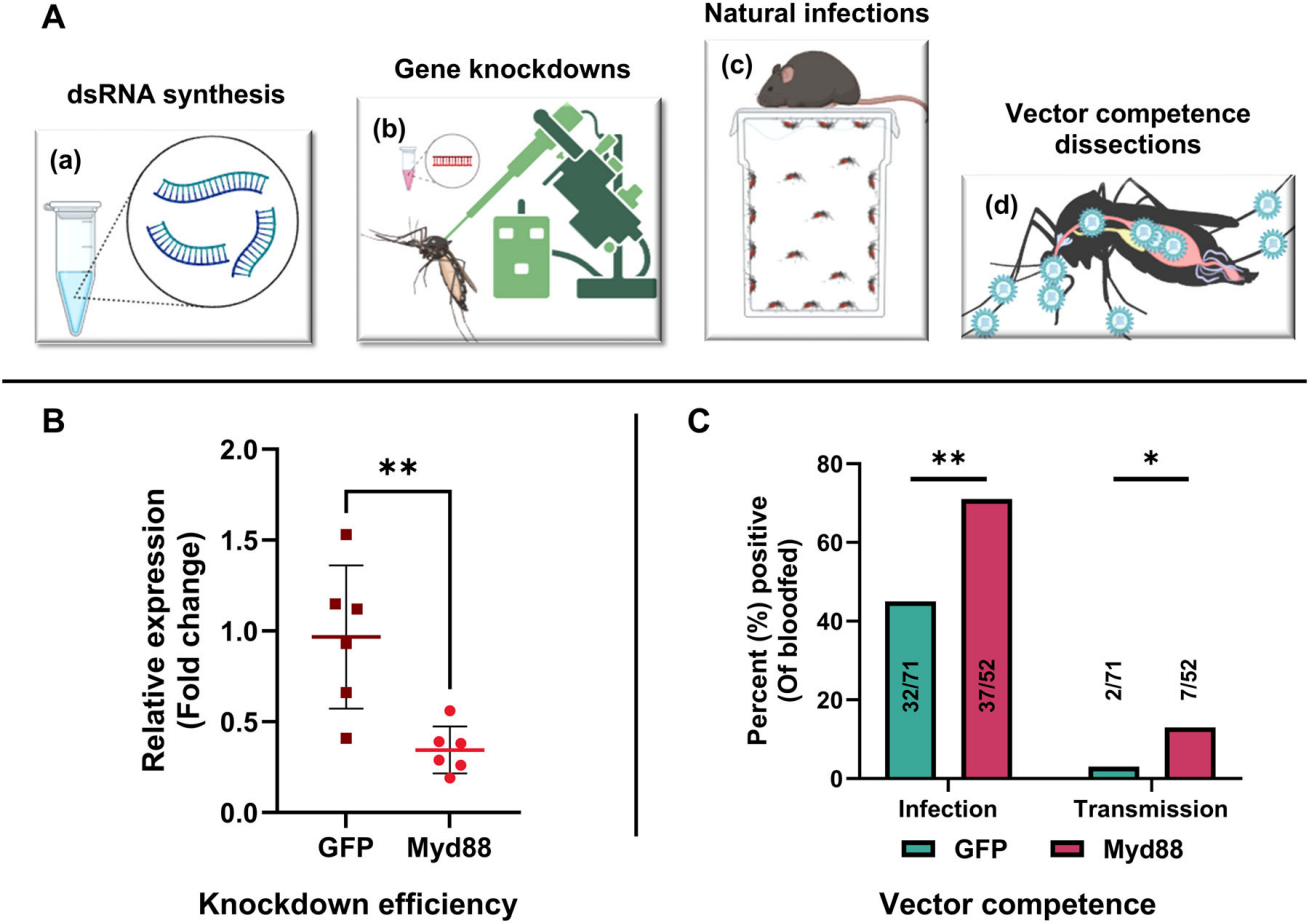
Knockdown of the Toll pathway adapter protein, Myd88 increases the vector competence of *Aedes aegypti* mosquitoes exposed to MAYV‐infected obese mice. (A) Experimental design. One to two days old *Ae. aegypti* females were coldly anesthetized and intrathoracically (IT) injected with 200 ng dsRNA against Myd88 or green fluorescent protein (GFP control). Three‐days‐post‐IT (dpIT) injections, mosquitoes (*n* = 60/mouse) were allowed to feed on obese mice exposed to MAYV (2 days‐postinfection) and 9 days later, mosquito bodies and saliva were collected and tested for infection and transmission rates respectively. (B) Knockdown efficiency was calculated by performing RT‐qPCR on the lysates collected from whole mosquito bodies 3‐days‐post‐IT injections. *C*_t_ values for Myd88 was normalized to the housekeeping gene (*Rp49*), and its expression was compared between GFP and Myd88 silenced mosquitoes using delta−delta *C*_t_ (ΔΔ*C*_t_) method for relative gene expression. Statistical analysis was done by unpaired *t*‐test. (C) Vector competence. Infection and transmission rates were measured between Myd88 and GFP knock‐down groups by calculating the percentage of MAYV‐positive bodies and saliva, respectively, compared to the total number of blood‐fed mosquitoes. Statistical analysis was conducted using a two‐sided Fisher's exact test. The level of significance is represented as follows: *p < 0.01; **p = 0.01 to 0.001. The error bars indicate the standard deviation (SD) of the mean.

To further confirm the role of the Toll pathway in controlling alphavirus infection in mosquitoes fed on obese mice, mosquitoes knocked down for Myd88, the adapter protein for the Toll pathway, or GFP as a knockdown control, were allowed to feed on MAYV‐infected obese mice at peak viremia (Figure 4A). We observed a knockdown efficiency of ~65% in mosquitoes knocked down for Myd88 compared to the GFP knockdown group (Figure 4B; **p = 0.0044) and significantly higher infection and transmission rates (Figure 4C; **p = 0.0056 and *p = 0.0353, respectively) in Myd88 knockdown group compared to the controls. These results validate our previous findings where artificial infection of mosquitoes knocked down for Myd88 showed increased transmission of CHIKV by *Ae. aegypti* mosquitoes [35], suggesting that the Toll pathway is important in controlling alphavirus infection of *Ae. aegypti* mosquitoes.

### 3.4 *Ae. aegypti* Mosquitoes Exposed to an Alphavirus‐Infected Obese Bloodmeal Show Altered Gene and Pathway Expression Which Differ Depending on the Virus and Time Postexposure

**Figure 5:**
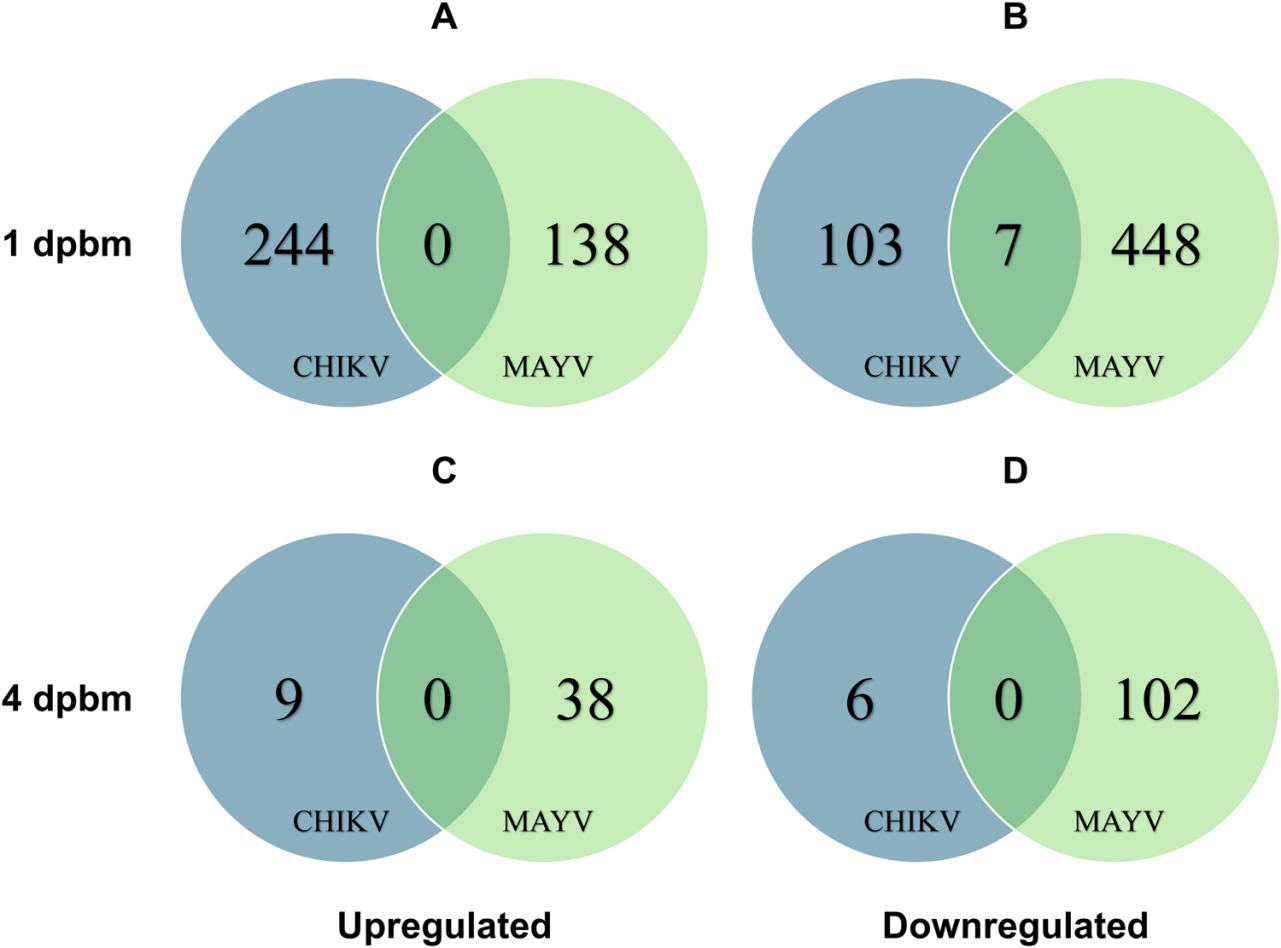
Identification of genes commonly expressed in mosquitoes fed on CHIKV‐ or MAYV‐infected obese versus lean mice at 1‐ and 4 days‐post‐infectious bloodmeals (dpbm). RNA sequencing (RNA‐seq) analysis was performed on the RNA extracted from mosquito midguts fed on CHIKV‐ or MAYV‐infected lean or obese mice at 1 and 4‐dpbm. Differentially expressed genes (DEGs) in obese versus lean‐fed mosquitoes with fold change > 3 were generated by integrated Differential Expression and Pathway (iDEP 1.1) analysis tool and selected for downstream analyses. Venn diagrams were generated using Venny 2.1 for commonly upregulated and downregulated genes expressed between mosquitoes fed on obese versus lean mice for CHIKV and MAYV at 1‐dpbm (A, B) and 4‐dpbm (C, D), respectively.

**Figure 6:**
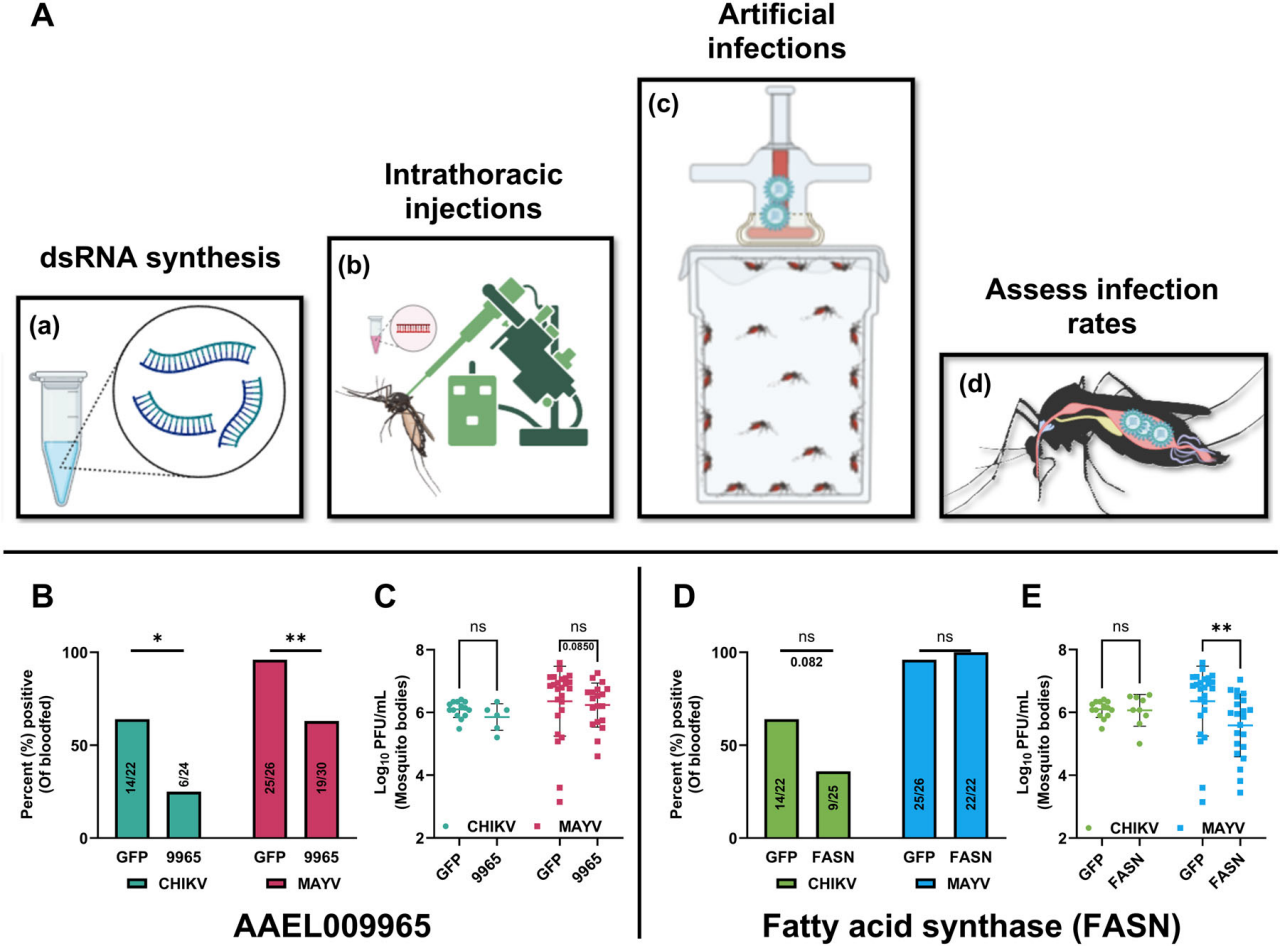
AAEL009965 and fatty acid synthase (FASN) are proviral for CHIKV and MAYV. (A) Experimental design. dsRNA was synthesized for AAEL009965 and FASN as GOI and green fluorescent protein (GFP) as control (a). One to two days old *Ae. aegypti* females were coldly anesthetized and intrathoracically (IT) injected with 500 ng dsRNA against GOI or control (b). Three‐days‐post‐IT injections, mosquitoes were exposed to 7 × 10^7^ PFUs/mL of CHIKV or MAYV using Hemotek feeders (c) and 7 days later, infection rates were calculated by calculating the percentage of bodies positive for CHIKV or MAYV compared to the total number of blood‐fed mosquitoes (d). Infection rates for CHIKV and MAYV after knockdown of AAEL009965 (B) or FASN (D) compared to GFP control. Statistical analysis was conducted using a two‐sided Fisher's exact test. Viral titers for CHIKV and MAYV after knockdown of AAEL009965 (C) or FASN (E). Statistical analysis was done by unpaired *t*‐test or Mann−Whitney test on log‐transformed titers tested for normality and outliers. The level of significance is represented as follows: *p < 0.01; **p = 0.01 to 0.001; ns, nonsignificant. The error bars indicate the standard deviation (SD) of the mean. These studies were performed as two independent biological replicates, which have been combined for analysis.

We compared the DEGs in mosquitoes exposed to CHIKV‐ or MAYV‐infected obese mice compared to mosquitoes that fed on lean counterparts at 1‐ and 4‐dpbm using RNA‐seq. At 1‐dpbm, 244 genes were upregulated (Figure 5A) and 110 (Figure 5B) were downregulated; at 4‐dpbm, we observed 9 upregulated (Figures 5C) and 6 downregulated (Figure 5D) genes for CHIKV. For MAYV, at 1‐dpbm, 138 genes were upregulated (Figure 5A) and 455 were downregulated (Figure 5B); at 4‐dpbm, 38 genes were upregulated (Figure 5C) and 102 were downregulated (Figure 5D). We next sought to identify genes that were consistently altered (up or downregulated) at the same time points for both CHIKV and MAYV. We found 7 genes downregulated at 1‐dpbm in mosquitoes fed on both CHIKV‐ and MAYV‐infected obese mice, with no genes found to be commonly upregulated (Figure 5C and Table 1). These genes were selected for further validation to determine their role in the infection of *Ae. aegypti* mosquitoes with alphaviruses. We then sought to broaden our search by identifying pathways altered in the same direction and same time point in response to both CHIKV‐ and MAYV‐infected obese bloodmeal, but there were no common pathways in response to both the viruses (Supporting Information S1: Figure S2).

**Table 1:** Downregulated genes common between mosquitoes exposed to either CHIKV‐ or MAYV‐infected obese bloodmeal at 1‐day‐post‐bloodmeal (dpbm).

		Log_2_ fold change	
Gene ID	Gene name	CHIKV	MAYV
AAEL007283	Acetyl CoA synthetase	−2.290126868	−1.88926
AAEL009965	Unspecified product	−2.184179696	−2.48433
AAEL010150	Unspecified product	−3.280313661	−2.02079
AAEL017536	Holotricin glycine‐rich repeat protein antimicrobial peptide	−1.856474924	−1.68861
AAEL022809	Unspecified product	−2.605221966	−2.66772
AAEL026093	Unspecified product	−5.955726837	−2.84262
AAEL029069	Fatty acid synthase	−4.356104755	−2.45794

### 3.5 An Unspecified Protein in *Ae. aegypti*, AAEL009965, and FASN Are Potentially Proviral for CHIKV and MAYV

We next sought to confirm the impact of the 7 commonly downregulated genes on the infection of *Ae. aegypti* mosquitoes with CHIKV and MAYV by performing gene knockdowns, followed by artificial infections with CHIKV or MAYV (Figure 6A). We hypothesized that silencing these genes, which were downregulated in mosquitoes fed on obese mice, would reduce the infection rates and/or viral titers. Mosquitoes knocked down for GOI or GFP were exposed to an infectious bloodmeal containing CHIKV 3‐dpIT injection. Knockdown of AAEL009965, an uncharacterized protein, and FASN showed a trend toward reduced CHIKV infection (Figure 6B,D; *p = 0.0163 and 0.0820, respectively), aligning with our RNA‐seq results. In contrast, the knockdown of AAEL007283, AAEL017536, and AAEL022809 showed a trend toward increased CHIKV infection. AAEL010150 and AAEL026093 were not tested as we failed to generate PCR amplicons to these genes for dsRNA synthesis. We then silenced AAEL009965 (labeled as 9965 in Figure 6) and FASN before exposure to an artificial blood meal containing MAYV. Knockdown of AAEL009965 showed reduced infection rates for MAYV (Figure 6B; **p = 0.0031), while knocking down FASN showed no difference compared to the control group (Figure 6D). We observed a knockdown efficiency of 60%−70% for both the genes for both CHIKV and MAYV (Supporting Information S1: Figure S3). We then sought to determine if knocking down these genes would impact viral titers. We found no difference between AAEL009965 or FASN and the control group for CHIKV (Figure 6C,E). However, with MAYV, we saw a trend toward reduced viral titers in the AAEL00965 group (Figure 6C; *p* = 0.0850) and significantly lower titers for the FASN knocked dwn group (Figure 6D; **p = 0.0093) compared to the control group.

## 4 Discussion

Vector‐borne diseases account for ~17% of infectious disease burden and more than 700 000 deaths annually [53], with mosquito‐borne infectious diseases attributing to the highest number of reported cases, mortality and economic losses in terms of disability‐adjusted life years (DALY) [54]. Several mosquito‐borne viruses of grave public health concern, like DENV [55], Zika virus (ZIKV) [56], and CHIKV [57] are globally transmitted by *Aedes* spp. mosquitoes [58]. The global presence of competent vectors, facilitated by climate change [59] and global travel [60], underscores the importance of developing novel transmission control strategies to prevent the spread of mosquito‐borne viruses. Although significant research has been conducted to elucidate the impact of mosquitoes on the transmission of these viruses, research on the effects of host biological factors on viral transmission by mosquitoes is limited.

Obesity is an important biological factor that has assumed the status of a pandemic [61], affecting 13% of adults globally and 42% of adults in the United States [23, 24], with a prediction that 50% of adults in the United States will be obese by 2030 [62]. This is concerning because, in the tropical and subtropical countries at the highest risk of mosquito‐borne diseases [63, 64], obesity levels are fast approaching those observed in developed nations [65]. Obesity is associated with several metabolic alterations in the host, including glucose, lipids [66], insulin [67], adipokines, cytokines, and chemokines [68]. The altered homeostasis of various factors in an obese host predisposes them to worse disease outcomes following infection with various respiratory [25, 26] and mosquito‐borne viruses [28, 69], including alphaviruses [27]. However, in the context of alphavirus transmission by *Ae. aegypti* mosquitoes, we found significantly lower infection and transmission rates in mosquitoes fed on obese compared to lean mice (Figure 2A,B), despite similar viremia in both the groups (Figure 2C,D), suggesting that obesity, but not viremia, was driving the reduced transmission potential.

Certain metabolic factors altered in an obese host have been shown to inhibit the replication of some mosquito‐borne viruses in mosquitoes. For example, mammalian insulin has previously been shown to inhibit flavivirus replication in mosquito cells and live mosquitoes by activating JAK/STAT [36] or extracellular‐signal‐related kinase (ERK) [70, 71] pathways. The ERK pathway, in turn, is triggered by the secretion of antimicrobial peptides (AMPs) [70, 71], which are essential components of the Toll and IMD pathway in insects [72]. The Toll pathway is critical in controlling DENV [47], and as we have shown, CHIKV [73] infection in *Ae. aegypti* mosquitoes. Obese hosts also show dysregulated lipid metabolism [66] and low‐density lipoproteins (LDLs) have been shown to inhibit flavivirus infection in mosquitoes [74]. Enrichment of lipid droplets was observed in mosquito midguts infected with the model alphavirus, Sindbis virus (SINV), and was also linked to activation of the Toll and IMD pathways [75], suggesting a possible role of lipids in activating the vector's immune response against viruses. Therefore, the impaired metabolic status of an obese host can potentially impact the transmission of mosquito‐borne viruses by activating various immune pathways in the mosquitoes. Since the midgut is the initial site of arbovirus infection [76] and a major transmission barrier [77], we evaluated midguts at 1‐ and 4‐dpbm representing transcriptional responses during midgut infection and escape, respectively [78, 79].

RT‐qPCR analyses on the RNA samples collected from mosquito midguts exposed to CHIKV‐ or MAYV‐infected lean and obese mice, tested for major known immune pathways in mosquitoes (RNAi, JAK/STAT, and Toll & IMD pathways), showed enrichment of genes involved in the Toll pathway in response to both CHIKV‐ or MAYV‐infected obese bloodmeals (Figure 3E,F). We previously showed that knockdown of the Toll pathway adapter protein, Myd88, reduced CHIKV infection in live *Ae. aegypti* mosquitoes [35]; however, we did not show the implication of Toll pathway knockdown in the context of a natural transmission cycle. Here we show that mosquitoes knocked down for Myd88 had significantly higher infection and transmission rates when exposed to MAYV‐infected obese mice compared to the GFP knocked‐down group (Figure 4C), underscoring the importance of the Toll pathway in controlling alphavirus infection in mosquitoes.

Since the objective of this study was to develop broadly effective transmission control strategies against mosquito‐borne viruses, we next sought to identify common genes and pathways with putative immune‐related functions that are altered in mosquitoes in response to both CHIKV‐ and MAYV‐infected obese bloodmeal at the same time‐point. RNA‐seq on midguts from mosquitoes fed on CHIKV‐ or MAYV‐infected lean or obese mice revealed 7 commonly downregulated genes in mosquito midguts at 1‐dpbm in response to both CHIKV‐ or MAYV‐infected obese blood (Figure 5), of which two genes: AAEL009965 and FASN showed antiviral activity against both CHIKV and MAYV after gene knockdowns (Figure 6). AAEL009965 is an ortholog of the tetratricopeptide repeat protein 12‐like in *Ae. albopictus* C6/36 cell line [80] and has been shown to be proviral for H7N9 avian influenza virus [81]; however, its role in insect immunity has not been studied yet. FASN is a key component in the lipid metabolism pathway, and its silencing in insects was shown to alter various key genes involved in fatty acid synthesis or lipogenesis [82]. Lipids play a crucial role in multiple stages of the alphavirus life cycle [83] and inhibition of FASN has been shown to impair DENV replication [84]. Moreover, in mosquitoes, fatty acids have been shown to impact energy metabolism, innate immunity, development, and reproduction [85]. These findings underscore the potential of using these genes as targets for controlling alphavirus transmission, pending further rigorous validation with other medically relevant alphaviruses or other arboviruses.

## 5 Conclusion

Mosquito‐borne viruses represent a significant public health threat and are responsible for substantial economic losses worldwide. For effective transmission, these viruses need to replicate in a vertebrate host and a vector, which imposes bottlenecks that impact their adaptability and transmission potential [86, 87]. Understanding the interactions between the virus, vector, and vertebrate host can provide valuable insights for designing transmission control strategies. In this study, we explored the role of host obesity—a biological variable—in modulating the mosquito immune response and identified specific genes that influence alphavirus infection rates in mosquitoes. Our findings demonstrate that silencing certain genes significantly impacts alphavirus infection and transmission by mosquitoes. This insight offers potential strategies for controlling the spread of mosquito‐borne diseases, such as developing and releasing transgenic mosquitoes deficient in these key genes; however, future studies are needed to confirm the efficacy of this strategy.

## Author Contributions

**Pallavi Rai:** conceptualization, data curation, investigation, methodology, validation, formal analysis, writing–original draft, visualization. **Emily M. Webb:** methodology, data curation, investigation, writing–review and editing. **Sally L. Paulson:** methodology, writing–review and editing. **Lin Kang:** software, data curation, writing–review and editing. **James Weger‐Lucarelli:** conceptualization, formal analysis, supervision, project administration, resources, writing–review and editing, writing–original draft, visualization.

## Conflicts of Interest

The authors declare no conflicts of interest.

## Supporting information

Supporting information.

## Data Availability

The data that support the findings of this study are available from the corresponding author upon reasonable request.
