# Supplementary material for: Obesity's Unexpected Influence: Reduced Alphavirus Transmission and Altered Immune Activation in the Vector
Source: J Med Virol. 2024 Oct 28;96(11):e70032. doi: 10.1002/jmv.70032 (PMC11600488; doi:10.1002/jmv.70032)
Supplement: Supplementary file 1 — Supporting information. [file JMV-96-e70032-s001.docx]

**SUPPLEMENTARY TABLES**

**Supplementary Table S1: List of *Ae. aegypti* primers used for RT-qPCR.**

| **Primers** | **Sequence** | **Reference** |
| --- | --- | --- |
| Dome-qPCR-F^1^ | AAACGGTGGCAAAATGAACT | *Jupatanakul et al., 2017* |
| Dome-qPCR-R^2^ | CTCCAGACCGGTGAGATTGT |  |
| Stat-qPCR-F | ACCGGACCTTCACCTTCTG | Rai et al., 2023 |
| Stat-qPCR-R | CCAGCTCACTGTTCGGAGAA |  |
| IMD-qPCR-F | ATGGAAGCATTTTGCGAGGGT |  |
| IMD-qPCR-R | CATTTTGCCGAGCGTTGGTT |  |
| Rel2-qPCR-F | TTTGAATGTGCTGTTGGGTC |  |
| Rel2- qPCR -R | GAATGTTGTTTCCGTGCTTA |  |
| Myd88- qPCR -F | CGATGCGTTCATTTTGTTTG |  |
| Myd88- qPCR -R | CACCGCTCAGAAATCAGCTT |  |
| Cactus- qPCR -F | AGACAGCCGCACCTTCGATTCC |  |
| Cactus- qPCR -R | CGCTTCGGTAGCCTCGTGGAT |  |
| Ago2- qPCR -F | GGCTGCTCACCCAATGTATCAAGA |  |
| Ago2- qPCR -R | AACCGTTCGTTTTGGCGTTGAT |  |
| Rp49-qPCR-F | AAGAAGCGGACGAAGAAGT |  |
| Rp49-qPCR-R | CCGTAACCGATGTTTGGC |  |
| AAEL009965 qPCR-F | CCAGTGGCGATAAGGGCAA | This study |
| AAEL009965 qPCR-R | TTCTCCAGGTCCTTCTTGTCC | This study |
| AAEL029069 qPCR-F | GCAGCCTACTCCTGGTCATC | This study |
| AAEL029069 qPCR-R | GCAACCACAGGAATGGCATC | This study |

**F^1^ stands for forward primer; R^2^ stands for reverse primer.

**Supplementary Table S2: List of *Ae. aegypti* primers used for dsRNA synthesis.**

| **Primers** | **Sequence** | **Reference** |
| --- | --- | --- |
| AAEL009965 T7-F^1^ | TAATACGACTCACTATAGGGGAACCAGGAGCGAACCGTTA | This study |
| AAEL009965 T7-R^2^ | TAATACGACTCACTATAGGGTTGTCGTCGCATCCTTCCC | This study |
| AAEL029069 T7-F | TAATACGACTCACTATAGGGGCAAGTGCTGTACCAATCCG | This study |
| AAEL029069 T7-R | TAATACGACTCACTATAGGGCAGCGGGTTCAGGAGTTCTT | This study |
| Myd88-T7-F  Myd88-T7-R | TAATACGACTCACTATAGGGGGCGATTGGTGGTTGTTATT  TAATACGACTCACTATAGGGTTGAGCGCATTGCTAACATC | Xi et al., 2008 |
| GFP-T7-F | TAATACGACTCACTATAGGGGACGTAAACGGCCACAAGTT | Rai et al., 2023 |
| GFP-T7-R | TAATACGACTCACTATAGGGTGTTCTGCTGGTAGTGGTCG |  |
|  |  |  |

**F^1^ stands for forward primer; R^2^ stands for reverse primer.

**SUPPLEMENTARY FIGURES**

**Figure S1: Testing mosquito midguts for RNA interference (RNAi) in response to alphavirus-infected obese bloodmeal.**

*Ae. aegypti* mosquitoes (n=50-60/mouse) were allowed to feed on CHIKV or MAYV-infected lean and obese mice, at peak viremia. Fully engorged mosquitoes were sorted and maintained in an environmental chamber at 28°C with 70-80% relative humidity, for 9 days. Midguts were dissected 1 day later, RNA was extracted, and gene expression was assessed by reverse-transcription quantitative PCR (RT-qPCR). Expression of genes of interest was normalized to the housekeeping gene, Rp49, and then compared between mosquitoes fed on CHIKV- or MAYV-infected lean or obese mice. **(A & B) RNAi pathway.** Ago2, a critical component of the RNA-induced silencing complex (RISC) in the RNAi pathway was tested for mosquitoes fed on CHIKV (A) or MAYV (B) infected lean or obese mice. Statistical analysis was done by unpaired t-test or Mann-Whitney test. The level of significance is represented as follows: ns – non-significant. The error bars indicate the standard deviation (SD) of the mean.

**Figure S2: Pathways altered in mosquitoes fed on CHIKV- or MAYV-infected obese vs lean mice at 1- and 4-days-post-infectious bloodmeals (dpbm).**

Differentially expressed genes (DEGs) in obese vs lean-fed mosquitoes with fold change > 3 were selected and pathways were generated by Gene Set Enrichment Analysis (GSEA) using Kyoto Encyclopedia of Genes and Genomes (KEGG) in iDEP 1.1. **(A & B) Pathway analysis at 1 day-post-infectious bloodmeal (dpbm)** for CHIKV (A) and MAYV (B) respectively. **(C & D) Pathway analysis at 4-dpbm** for CHIKV (C) and MAYV (D) respectively. Green dots indicate downregulated pathways, and red dots indicate upregulated pathways in mosquitoes fed on CHIKV- or MAYV-infected obese vs lean mice.

**Figure S3: Knockdown efficiency for AAEL009965 and fatty acid synthase.**

RT-qPCR was performed on lysates collected from 5 whole mosquitoes at 3 days post-IT injection. Ct values for the genes of interest (GOI), AAEL009965 **(A)** and FASN **(B)** were normalized to the housekeeping gene (*Rp49*), and their expression was compared between GFP and AAEL009965 or FASN silenced mosquitoes, using delta delta Ct (ΔΔCt) method for relative gene expression. Statistical analysis was done by Mann-Whitney test; ***p = 0.001 to 0.0001. Data are presented as mean with 95% confidence interval (CI). The error bars indicate the standard deviation (SD) of the mean.
